# More Than Five Years of Sustained Remission With Mepolizumab in Adolescent-Onset Eosinophilic Granulomatosis With Polyangiitis: A Case Report

**DOI:** 10.7759/cureus.99496

**Published:** 2025-12-17

**Authors:** Tomoyuki Araya, Toshiyuki Kita, Tasuku Iwabuchi, Takayuki Higashi, Ryo Hara

**Affiliations:** 1 Respiratory Medicine, National Hospital Organization (NHO) Kanazawa Medical Center, Kanazawa, JPN

**Keywords:** adolescent and young adults, anca-negative vasculitis, eosinophilic granulomatosis with polyangiitis (egpa), long-term remission, mepolizumab

## Abstract

Eosinophilic granulomatosis with polyangiitis (EGPA) typically affects middle-aged adults, and adolescent-onset disease is rare and scarcely documented in long-term real-world follow-up. We describe an 18-year-old woman with asthma who presented with fever, muscle pain, wet cough, and dyspnea. Laboratory tests showed marked leukocytosis (27,600/µL) with severe eosinophilia (17,940/µL), elevated creatine kinase, normal Krebs von den Lungen-6, elevated surfactant protein-D, markedly elevated serum immunoglobulin E, and negative proteinase 3- and myeloperoxidase-anti-neutrophil cytoplasmic antibody. Imaging revealed bilateral patchy infiltrates, and transbronchial lung biopsy demonstrated eosinophilic infiltration with granuloma formation and features of vasculitis, establishing the diagnosis of EGPA. Prednisolone 25 mg/day induced rapid improvement with normalization of eosinophils by day 12. She remained stable for nearly two years on low-dose prednisolone until asymptomatic eosinophilia recurred during tapering. Mepolizumab 300 mg every four weeks was then initiated, leading to immediate eosinophil normalization. Prednisolone was discontinued three years and 10 months after starting mepolizumab, and she has maintained remission for more than five years under continued therapy. This real-world case shows that, in adolescent-onset EGPA, where corticosteroids alone controlled disease for only two years, mepolizumab achieved sustained remission without significant adverse effects and enabled complete steroid withdrawal, underscoring its value as a long-term steroid-sparing strategy.

## Introduction

Eosinophilic granulomatosis with polyangiitis (EGPA) is a rare systemic vasculitis characterized by asthma, blood and tissue eosinophilia, extravascular granuloma formation, and small-vessel vasculitis [1]. Two major clinicopathologic subsets are recognized: an anti-neutrophil cytoplasmic antibody (ANCA)-positive form with predominant vasculitic manifestations and an ANCA-negative form with prominent eosinophil-mediated organ involvement. The disease primarily affects adults, most commonly those in their 40s to 60s, and adolescent-onset EGPA is exceedingly uncommon [2]. Although overall survival is generally favorable, the five-year relapse risk remains high at 35-46%, underscoring the need for effective long-term disease control [2].

Because young patients may suffer significant lifelong consequences from chronic corticosteroid exposure, establishing effective steroid-sparing strategies is crucial. Recent evidence supports the use of mepolizumab, an anti- interleukin-5 (IL-5) monoclonal antibody, in reducing relapse rates and corticosteroid requirements [3]. Mepolizumab was approved for EGPA in 2017 and has since become an important therapeutic option for relapsing or refractory disease. However, long-term real-world outcomes, especially in young-onset cases, remain limited.

We report a woman who developed eosinophilic granulomatosis with polyangiitis at age 18 and experienced disease recurrence after two years of corticosteroid therapy, but subsequently achieved more than five years of remission and successful steroid discontinuation with mepolizumab.

## Case presentation

An 18-year-old female student with a known history of bronchial asthma, diagnosed at age 17 and well controlled with budesonide/formoterol and montelukast, presented with new-onset muscle pain involving the scapular region, upper limbs, and lower back, along with wet cough, wheezing, low-grade fever, and exertional dyspnea that limited her daily activities. She had no history of smoking, dust exposure, pet ownership, food or drug allergies, or relevant family history. Her asthma had remained stable on regular outpatient follow-up every two months.

**Figure 1:**
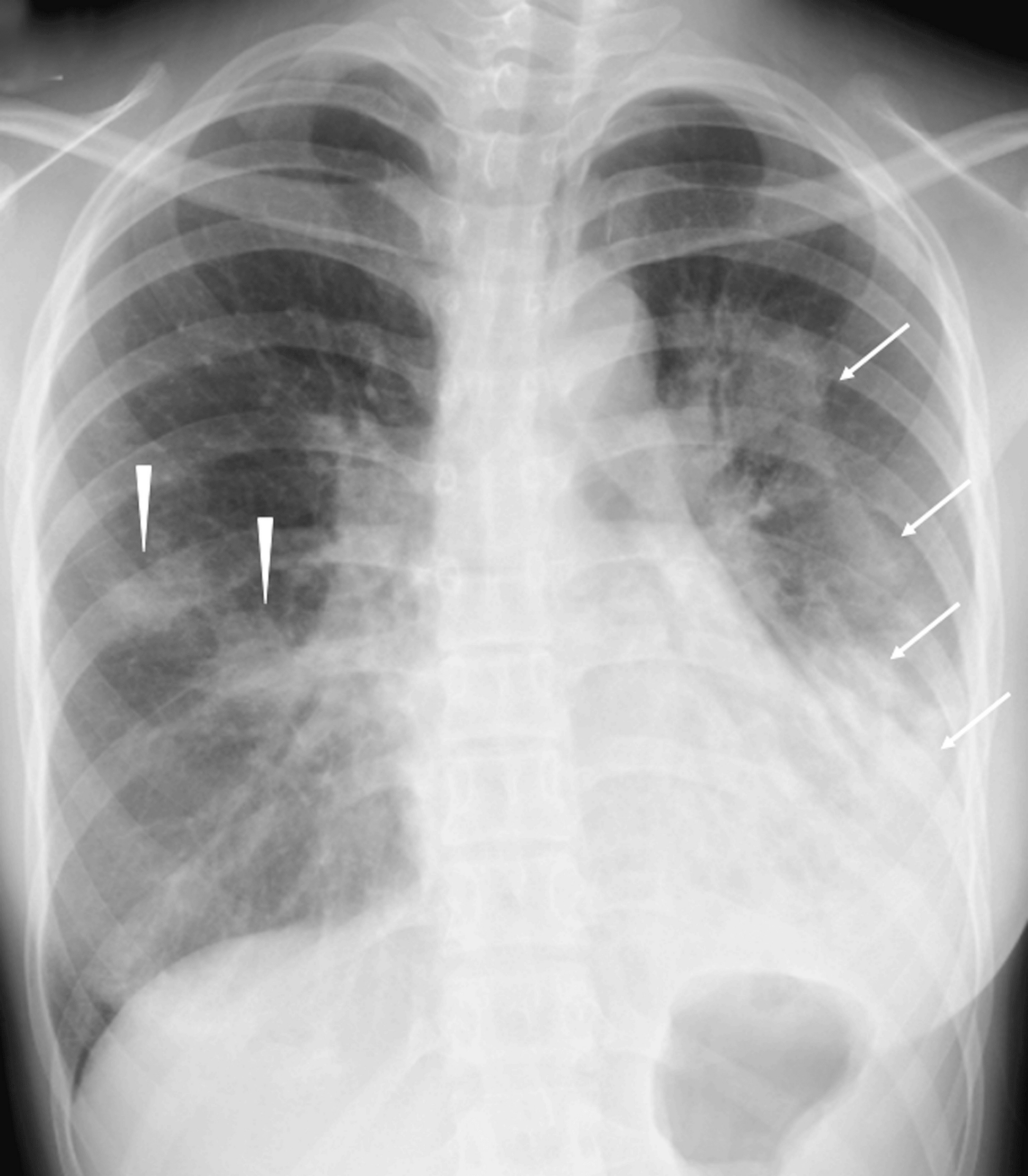
Initial Chest X-ray Showing Bilateral Pulmonary Infiltrates Chest X-ray at presentation demonstrating patchy opacities in the right mid lung field (arrowheads) and extensive infiltrates in the left lung, predominantly in the mid-to-lower zones but extending into the upper zone (arrows).

On admission, her vital signs were: temperature 38.1°C, blood pressure 113/72 mmHg, heart rate 98/min, respiratory rate 16/min, and oxygen saturation 96% on room air. Chest auscultation revealed mild bilateral wheezes without crackles. Other physical examination findings included no abnormalities of the skin, eyes, or oral mucosa; no nasal bleeding or crust formation; normal heart sounds without murmurs; no abdominal organomegaly or tenderness; and no neurological deficits. Chest X-ray showed patchy opacities in the right mid lung field and extensive infiltrates in the left lung, predominantly in the mid-to-lower zones but extending into the upper zone (Figure 1).

**Figure 2:**
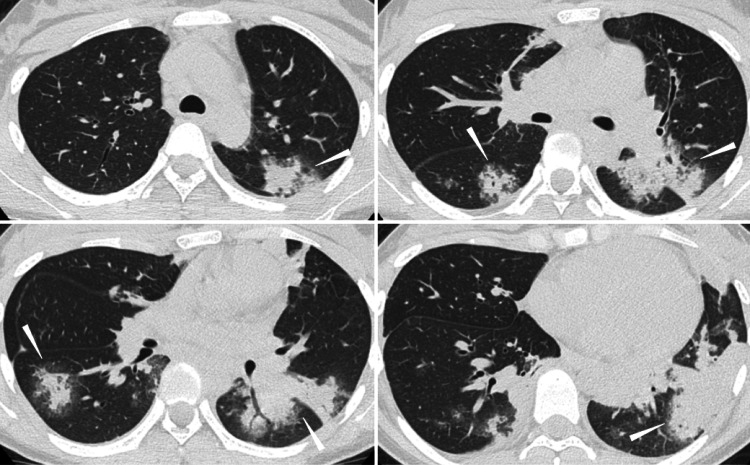
Chest Computed Tomography Showing Bilateral Patchy Opacities and Infiltrative Consolidations Axial chest computed tomography images demonstrating multiple bilateral patchy opacities and infiltrative consolidations (arrowheads) without mediastinal lymphadenopathy. Lesions are predominantly distributed in the lower lobes with varying degrees of peripheral and peribronchial involvement.

Chest computed tomography revealed multiple bilateral patchy opacities and infiltrative consolidations without mediastinal lymphadenopathy (Figure 2).

Laboratory studies demonstrated marked leukocytosis (27,600/µL) with pronounced eosinophilia (17,940/µL; 65%), elevated C-reactive protein (3.38 mg/dL), normal Krebs von den Lungen-6, elevated surfactant protein-D, markedly elevated serum immunoglobulin E (4,125 IU/mL), and elevated creatine kinase (358 U/L), with additional increases in creatine kinase-MB isoenzyme (57 U/L), B-type natriuretic peptide (137.3 pg/mL), and troponin I (4,861 pg/mL); proteinase 3-ANCA and myeloperoxidase-ANCA were both negative, arterial blood gas analysis showed mild hypoxemia (PaO₂ 77.7 torr), and urinalysis revealed no abnormalities (Table 1).

**Table 1:** Laboratory Findings on Admission WBC: white blood cell; Neu: neutrophil; Eos: eosinophil; Lym: lymphocyte; Mono: monocyte; Baso: basophil; RBC: red blood cell; Hb: hemoglobin; Hct: hematocrit; Plt: platelet; CRP: C-reactive protein; T-Bil: total bilirubin; TP: total protein; Alb: albumin; ALP: alkaline phosphatase; AST: aspartate aminotransferase; ALT: alanine aminotransferase; γ-GTP: gamma-glutamyl transpeptidase; LDH: lactate dehydrogenase; Na: sodium; K: potassium; Cl: chloride; BUN: blood urea nitrogen; Cr: creatinine; eGFR: estimated glomerular filtration rate; UA: uric acid; Amy: amylase; CK: creatine kinase; CK-MB: creatine kinase-MB isoenzyme; BNP: B-type natriuretic peptide; TnI: troponin I; HbA1c: hemoglobin A1c; ACE: angiotensin-converting enzyme; PT-INR: prothrombin time–international normalized ratio; IgG/IgA/IgM/IgE: immunoglobulin G/A/M/E; KL-6: Krebs von den Lungen-6; SP-D: surfactant protein-D; SP-A: surfactant protein-A; ANA: antinuclear antibody; RF: rheumatoid factor; PR3-ANCA: proteinase 3–ANCA; MPO-ANCA: myeloperoxidase–ANCA; Ab: antibody; CCP: cyclic citrullinated peptide; Sm: Smith; dsDNA: double-stranded DNA; RNP: ribonucleoprotein; Scl-70: topoisomerase I; RNAP III: RNA polymerase III; ARS: aminoacyl-tRNA synthetase; MDA5: melanoma differentiation-associated gene 5; IGRA: interferon-γ release assay; Ag: antigen; PaCO₂: partial pressure of carbon dioxide; PaO₂: partial pressure of oxygen; BE: base excess; SaO₂: arterial oxygen saturation.

Parameter (Unit)	Value	Reference
WBC (/µL)	27600	4500-9000
Neu (%)	24.0	38-74
Eos (%)	65	0-10
Lym (%)	9.0	16.5-49.5
Mono (%)	2.0	5-10
Baso (%)	0	0-2
RBC (×10⁴/µL)	447	382-500
Hb (g/dL)	13.2	11.7-14.6
Hct (%)	38.5	34.3-44.2
Plt (×10⁴/µL)	33.5	15.0-35.0
CRP (mg/dL)	3.38	0-0.4
T-Bil (mg/dL)	0.6	0.3-1.2
TP (g/dL)	6.5	6.7-8.3
Alb (g/dL)	3.2	4.0-5.0
ALP (U/L)	251	113-359
AST (U/L)	55	13-33
ALT (U/L)	47	6-27
γ-GTP (IU/L)	18	10-47
LDH (U/L)	492	119-229
Na (mEq/L)	137	135-149
K (mEq/L)	4.2	3.5-4.9
Cl (mEq/L)	102	96-108
BUN (mg/dL)	12.8	8-22
Cr (mg/dL)	0.72	0.5-0.8
eGFR (mL/min)	89.6	60-100
UA (mg/dL)	2.9	2.3-7.0
Amy (U/L)	43	35-140
Ferritin (ng/mL)	87	12-60
CK (U/L)	358	45-163
CK-MB (U/L)	57	0-25
BNP (pg/mL)	137.3	<18.4
TnI (pg/mL)	4861	<26.2
HbA1c (%)	5.0	<6.4
ACE (U/L)	7.7	7.7-29.4
PT-INR	1.12	0.8-1.2
D-dimer (µg/mL)	1.6	0-1
IgG (mg/dL)	1300	870-1700
IgA (mg/dL)	193	110-410
IgM (mg/dL)	146	35-220
IgE (IU/mL)	4125	<232
KL-6 (U/mL)	236	<500
SP-D (ng/mL)	203	<109.9
SP-A (ng/mL)	25.6	18.4-49
Immunological tests		
ANA titer	<40	<40
RF (IU/mL)	65	<20
PR3-ANCA (U/mL)	<1.0	<1.0
MPO-ANCA (U/mL)	<1.0	<1.0
Anti-CCP Ab (U/mL)	<0.5	<4.5
Anti-Sm Ab (U/mL)	<1.0	<10
Anti-dsDNA Ab (U/mL)	<10	<10
Anti-RNP Ab (U/mL)	<2.0	<10
Anti-SSA Ab (U/mL)	<1.0	<10
Anti-SSB Ab (U/mL)	<1.0	<10
Anti-Scl-70 Ab (U/mL)	1.5	<10
Anti-centromere Ab	<5	<10
Anti-RNAP III Ab	negative	negative
Anti-ARS Ab	negative	negative
Anti-MDA5 Ab	negative	negative
Microbiological tests		
β-D-glucan (pg/mL)	2.238	<11
IGRA	negative	negative
Anti-GPL core IgA (U/mL)	negative	negative
Streptococcus pneumoniae urinary Ag	negative	negative
Legionella urinary Ag	negative	negative
Mycoplasma Ag	negative	negative
Arterial blood gas analysis		
pH	7.457	7.35-7.45
PaCO2 (torr)	33.2	35-45
PaO2 (torr)	77.7	80-100
HCO3^-^ (mmol/L)	23.1	22-28
BE (mmol/L)	0.5	-2.4–2.4
SaO2 (%)	95.5	95-98
Lactate (mmol/L)	1.6	1-1.5

Electrocardiography revealed an RSR′-like pattern in leads V2-V4. Transthoracic echocardiography demonstrated a left ventricular ejection fraction of 56% (modified Simpson’s method) with mild hypokinesis of the anterior apical wall. Cardiac magnetic resonance imaging and myocardial scintigraphy using technetium-99m pyrophosphate and thallium-201 showed no significant abnormalities. Given the procedure's invasiveness and the limited diagnostic yield in this context, endomyocardial biopsy was not performed. Taken together with the laboratory findings, these results were interpreted by a cardiologist as indicating mild myocardial involvement.

**Figure 3:**
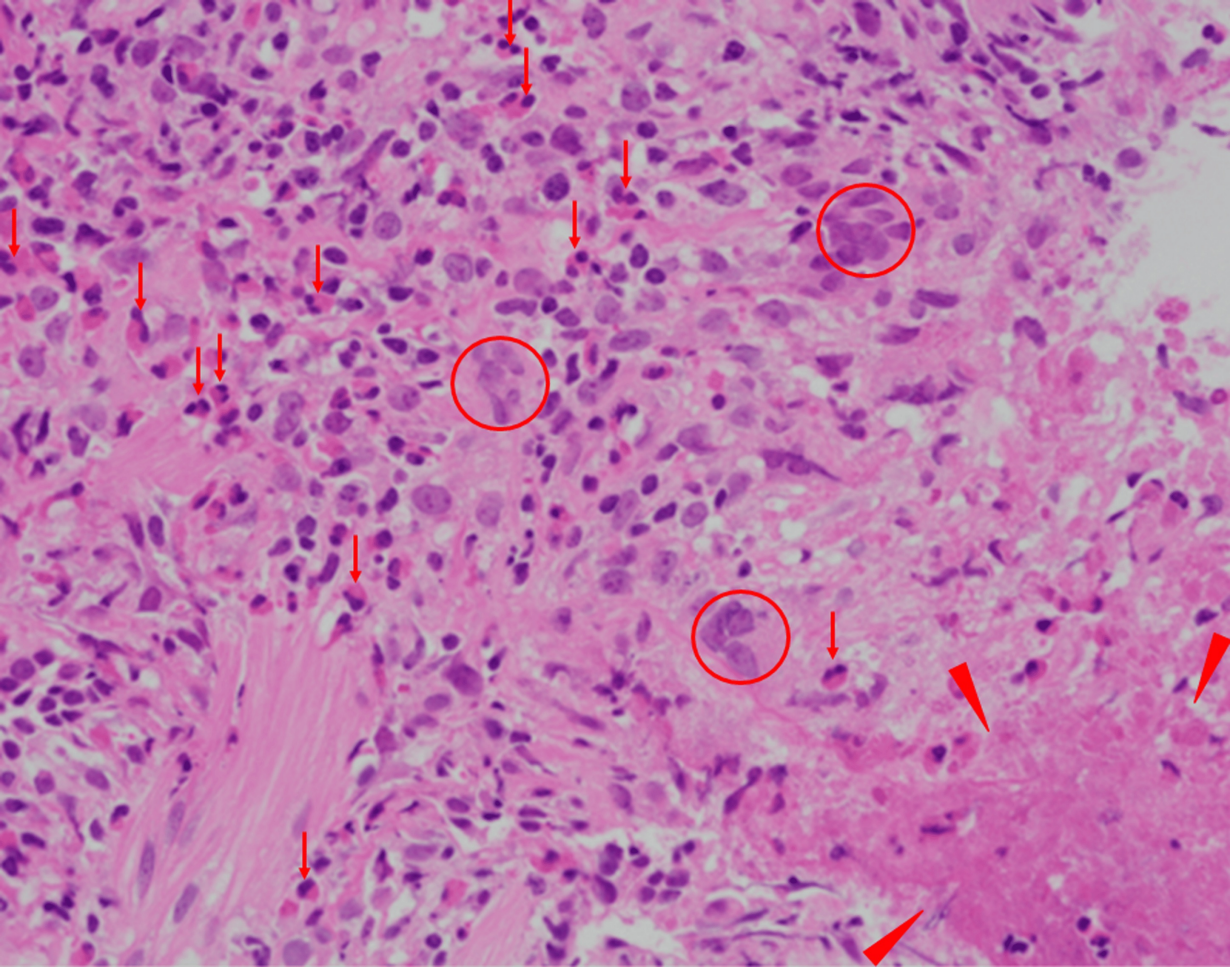
Histopathological Findings of the Transbronchial Lung Biopsy Transbronchial lung biopsy obtained from the left B8a segment using endobronchial ultrasonography with a guide sheath (EBUS-GS) revealed dense eosinophilic infiltration within the bronchiolar and alveolar tissue (arrows). Granuloma formation consisting of clusters of epithelioid cells was also observed (circles). Areas of necrosis suggestive of ischemic injury secondary to vasculitis were present (arrowheads). Hematoxylin and eosin stain, ×400.

Bronchoscopy revealed mild atrophic bronchial mucosa without endobronchial lesions. Bronchoalveolar lavage fluid (BALF) obtained from the left B5 bronchus showed a recovery rate of 74%, with a total cell count of 8.8 × 10⁵ cells. Differential cell counts revealed 68% eosinophils, 21% alveolar macrophages, 6% neutrophils, and 5% lymphocytes. The CD4/CD8 ratio was 2.3. Both bacterial and mycobacterial cultures of the BALF were negative, and cytology showed no malignant cells. Transbronchial lung biopsy was performed from the left B8a segment using endobronchial ultrasonography with a guide sheath (EBUS-GS). The biopsy specimens showed marked eosinophilic infiltration within bronchiolar and alveolar tissue, along with granuloma formation composed of clusters of epithelioid cells. The presence of necrosis suggested ischemic changes secondary to vasculitis (Figure 3).

These histopathological findings were consistent with eosinophilic granulomatosis with polyangiitis.

Additional organ involvement included eosinophilic gastroenteritis confirmed by gastric and duodenal biopsies, eosinophilic sinusitis with maxillary sinus mucosal thickening and biopsy-proven eosinophilic infiltration, and increased eosinophils without dysplasia on bone marrow biopsy, effectively excluding hematologic neoplasms. Nerve conduction studies were also performed and showed no evidence of conduction delay, indicating the absence of peripheral neuropathy.

**Figure 4:**
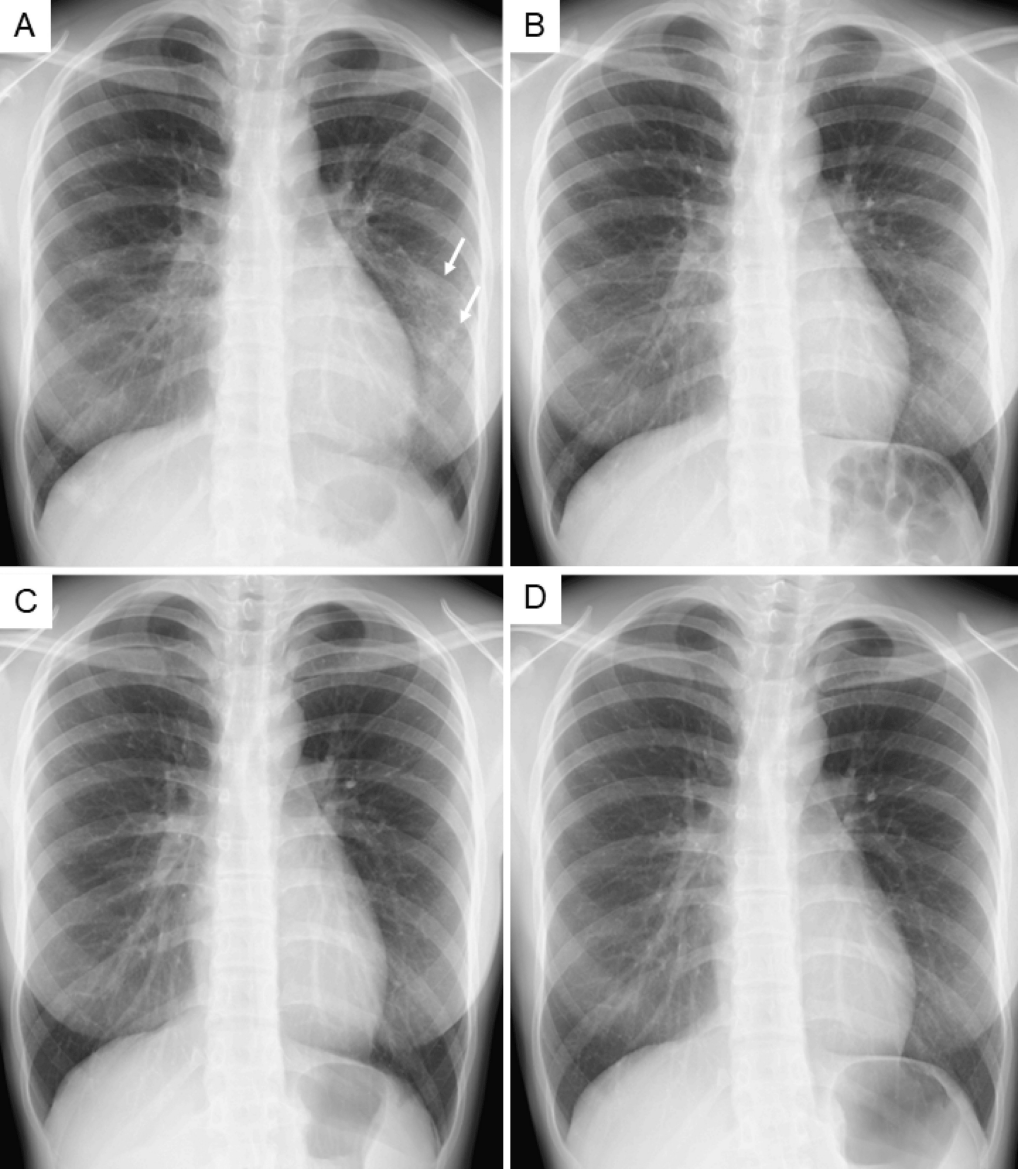
Serial Chest X-rays During Steroid Therapy and Long-term Mepolizumab Treatment (A) Chest X-ray on day 5 of prednisolone therapy showing marked improvement in bilateral infiltrates, with only faint residual opacities remaining in the left mid-to-lower lung zones (arrowheads). (B) Chest X-ray on day 12 demonstrating near-complete resolution of pulmonary infiltrates, corresponding to normalization of clinical symptoms and a Birmingham Vasculitis Activity Score of 0. (C) Chest X-ray at the time of eosinophilic granulomatosis with polyangiitis (EGPA) relapse, 1 year and 10 months after initiating prednisolone, showing no new infiltrates despite recurrent peripheral eosinophilia. (D) Chest X-ray at the most recent follow-up, 5 years and 1 month after starting mepolizumab, demonstrating sustained radiographic remission.

Based on the clinical triad of asthma, marked eosinophilia, and multisystem involvement, including pulmonary infiltrates with biopsy-proven eosinophilic granulomatous inflammation with necrosis suggestive of vasculitis, eosinophilic gastroenteritis, eosinophilic sinusitis, and mild myocardial involvement, the patient met the definite diagnostic criteria for eosinophilic granulomatosis with polyangiitis established by the Japanese Ministry of Health and Welfare in 1998 [4]. She also fulfilled four of the six 1990 American College of Rheumatology (ACR) classification criteria (asthma, sinus disease, peripheral eosinophilia, and pulmonary infiltrates), demonstrating diagnostic consistency with international standards [5]. Retrospective application of the 2022 American College of Rheumatology/European Alliance of Associations for Rheumatology (ACR/EULAR) classification criteria [6] yielded a score of 10 points (threshold ≥6), further reinforcing the diagnostic validity. The Birmingham Vasculitis Activity Score (BVAS) at presentation was 11, indicating moderate disease activity without major organ-threatening manifestations [7]. Although she had mild myocardial involvement and eosinophilic gastroenteritis, these did not meet the definitions of the cardiac or gastrointestinal items in the revised Five-Factor Score (FFS 2009) [8]; therefore, her FFS 2009 was 0, and treatment options were discussed in a multidisciplinary conference involving cardiology, gastroenterology, and otolaryngology. Through a shared decision-making process, systemic corticosteroid monotherapy was selected, which is consistent with contemporary international recommendations indicating that glucocorticoids alone are acceptable for FFS 0 disease without life-threatening organ involvement [9]. Immunosuppressive agents were not initiated, given her young age and the desire to avoid potential long-term adverse effects, including carcinogenic risk. Prednisolone was initiated at 25 mg/day, resulting in rapid clinical and laboratory improvement. Five days after treatment initiation, the white blood cell count decreased from 27,600/µL (eosinophils 17,940/µL) to 12,000/µL (eosinophils 1,560/µL), and by day 12 both values had normalized (WBC 8,000/µL; eosinophils 400/µL). Chest X-ray showed marked improvement in bilateral infiltrates by day 5 and near-complete resolution by day 12, at which time all symptoms had disappeared, and her Birmingham Vasculitis Activity Score (BVAS) had decreased to 0 (Figure 4).

Prednisolone was gradually tapered and reached a maintenance dose of 5 mg approximately 11 months after treatment initiation, during which she remained asymptomatic with no biochemical or radiographic abnormalities. After one year and 10 months of prednisolone therapy, recurrence of peripheral eosinophilia was detected (eosinophils 1,628/µL) despite the absence of symptoms and no new infiltrates on chest X-ray. Following discussion with the patient and her family, prednisolone was continued at the same dose, and humanized anti-IL-5 monoclonal antibody mepolizumab 300 mg every four weeks was introduced. Eosinophil counts normalized within one week after mepolizumab initiation and have since remained consistently low (10-30/µL), with no recurrence of symptoms, radiographic abnormalities, or cardiac dysfunction. The patient has now received mepolizumab for five years and one month, totaling 67 doses, with sustained disease control. Following the introduction of mepolizumab, prednisolone was slowly tapered and ultimately discontinued three years and ten months later, resulting in long-term steroid-free remission.

## Discussion

Adolescent-onset EGPA is exceedingly rare, and real-world longitudinal data extending over multiple years are almost nonexistent. Against this background, the present case provides important insight into the long-term treatment course, particularly regarding the durability of corticosteroid therapy and the sustained efficacy of mepolizumab. Although the patient exhibited multisystem involvement, including pulmonary and mild cardiac manifestations-her Five-Factor Score was 0, and corticosteroid monotherapy was selected through multidisciplinary discussion and shared decision-making. However, the steroid effect was not durable, with relapse occurring in less than two years.

Cardiac involvement is a major determinant of morbidity and mortality in EGPA, occurring in 27.7-43% of patients and accounting for nearly half of EGPA-related deaths [10,11]. Importantly, ANCA-negative EGPA, such as in this case, is strongly associated with eosinophil-mediated cardiac injury: only 14.9% of eosinophilic myocarditis cases are ANCA-positive, whereas 71.4% of EGPA patients who died from cardiac causes were ANCA-negative [11]. Genomic analyses further demonstrate that ANCA-negative EGPA exhibits IL-5-related pathogenic signatures, supporting the biological plausibility of interleukin-5-targeted therapy [11]. Cardiac involvement is also an independent predictor of mortality, with markedly lower five-year survival in affected patients [10]. Although advances in diagnostic and therapeutic strategies have reduced cardiac mortality in recent years, the optimal immunosuppressive regimen for cardiac EGPA remains under debate, and most reported cases still require high-dose glucocorticoids with cyclophosphamide [10,12]. Although minimizing lifelong exposure to both corticosteroids and immunosuppressive agents is particularly important in young patients, long-term evidence to guide clinical decisions in this population remains limited.

Given these considerations, the therapeutic role of IL-5 blockade is of particular interest. In the pivotal randomized controlled MIRRA trial, mepolizumab demonstrated substantial efficacy in patients with relapsing or refractory eosinophilic granulomatosis with polyangiitis, with 28% of treated participants achieving a cumulative remission duration of at least 24 weeks, highlighting its ability not only to induce remission but also to support sustained disease control [3]. Despite the robust evidence provided by the MIRRA trial, no studies have specifically evaluated long-term biologic therapy in adolescent-onset EGPA. The present case demonstrates more than five years of sustained remission and excellent safety under mepolizumab despite ANCA-negative disease and mild myocardial involvement-features typically associated with a need for more aggressive immunosuppression.

Regarding other IL-5-targeted biologics, a previous report described a 22-year-old patient with eosinophilic granulomatosis with polyangiitis who was successfully treated with benralizumab [13]. Similar to the present case, the patient was ANCA-negative and experienced recurrent cardiac involvement in the form of pericarditis. Benralizumab led to marked clinical improvement and complete steroid withdrawal, suggesting the potential effectiveness of IL-5 pathway blockade in this patient population. However, the observation period in that report was limited to eight months. In contrast, the present case demonstrates sustained remission for five years, indicating substantially greater real-world treatment durability. Notably, mepolizumab was approved for EGPA by the U.S. Food and Drug Administration (FDA) in 2017, whereas benralizumab received FDA approval for EGPA only recently in 2024. Thus, in the previously reported case as well as throughout the period before 2024, the use of benralizumab for EGPA remained entirely off-label. Because differences in therapeutic mechanisms between mepolizumab and benralizumab may result in differences in clinical response, accumulation of larger case series with long-term follow-up is needed to clarify their relative efficacy in young ANCA-negative EGPA. Importantly, this case highlights a practical treatment pathway for adolescent-onset EGPA, showing that corticosteroid monotherapy may be insufficient even in patients with a low Five-Factor Score, and that timely initiation of IL-5-targeted therapy can provide durable disease control. These observations may help guide therapeutic decision-making in similarly complex young patients, for whom long-term evidence remains scarce.

## Conclusions

This case illustrates that adolescent-onset eosinophilic granulomatosis with polyangiitis can initially respond to corticosteroid therapy yet relapse during tapering despite low disease severity and a Five-Factor Score of 0. Mepolizumab enabled rapid normalization of eosinophils, sustained remission for more than five years, and successful steroid discontinuation without adverse effects. This long-term real-world outcome highlights the value of anti-IL-5 biologic therapy as an effective and well-tolerated steroid-sparing strategy in young patients with EGPA.
